# Embedding Ultra-High-Molecular-Weight Polyethylene Fibers in 3D-Printed Polylactic Acid (PLA) Parts

**DOI:** 10.3390/polym11111825

**Published:** 2019-11-06

**Authors:** Cătălin Gheorghe Amza, Aurelian Zapciu, Arnheiður Eyþórsdóttir, Auðbjörg Björnsdóttir, Jonathan Borg

**Affiliations:** 1 University “Politehnica” of Bucharest, 060042 Bucharest, Romania; aurelianzapciu@yahoo.com; 2 University of Akureyri, Akureyri 600, Iceland; arnh@unak.is (A.E.); audbjorg@unak.is (A.B.); 3 University of Malta, Msida, MSD 2080, Malta; jonathan.borg@um.edu.mt

**Keywords:** additive manufacturing, fiber reinforced, UHMWPE, Dyneema

## Abstract

This study aims to assess whether ultra-high-molecular-weight polyethylene (UHMWPE) fibers can be successfully embedded in a polylactic acid (PLA) matrix in a material extrusion 3D printing (ME3DP) process, despite the apparent thermal incompatibility between the two materials. The work started with assessing the maximum PLA extrusion temperatures at which UHMWPE fibers withstand the 3D printing process without melting or severe degradation. After testing various fiber orientations and extrusion temperatures, it has been found that the maximum extrusion temperature depends on fiber orientation relative to extrusion pathing and varies between 175 °C and 185 °C at an ambient temperature of 25 °C. Multiple specimens with embedded strands of UHMWPE fibers have been 3D printed and following tensile strength tests on the fabricated specimens, it has been found that adding even a small number of fiber strands laid in the same direction as the load increased tensile strength by 12% to 23% depending on the raster angle, even when taking into account the decrease in tensile strength due to reduced performance of the PLA substrate caused by lower extrusion temperatures.

## 1. Introduction

**Figure 1:**
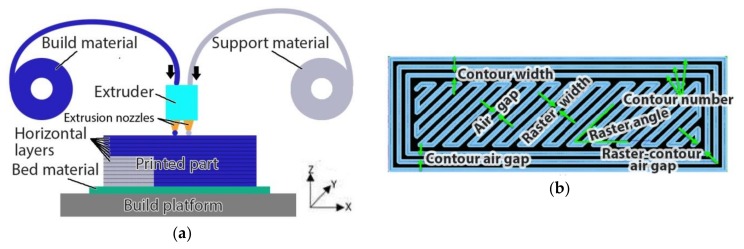
Material extrusion 3D printing: (**a**) Process schematic—the extruder and the build platform move relative to one another in the XY plane in order to deposit a horizontal layer and in the Z direction in order to position for the next layer; (**b**) horizontal layer elements.

Since 2009, when patents held by the American company, Stratasys, on fused deposition modeling 3D printing started to expire, this additive manufacturing (AM) process has seen extensive implementation under the recognized name of material extrusion 3D printing (ME3DP). Nearly a decade later, ME3DP has become one of the most widespread AM techniques [1] due to its relatively low cost in terms of hardware equipment and build materials and due to its ease of use. ME3DP is a process where parts are fabricated by depositing layers of material one on top of another, with each layer being made by extruding thermoplastic feedstock that comes in the form of filament or pellets along a predetermined path. The first layer is printed onto a platform which moves in the horizontal plane relative to the extruder. Upon finalizing the first horizontal layer, the extruder moves relative to the platform in a perpendicular direction and proceeds to deposit the next horizontal layer. A schematic of a 3D printer which works based on the ME3DP principle is shown in Figure 1a. The relative motion (extrusion path) of the system is determined based on several parameters, which include nozzle diameter, fill pattern and orientation, number of contours and their width, and gaps. The key elements that compose each individual horizontal layer are shown in Figure 1b.

As with all 3D printing processes, researchers have been continuously searching for new materials and methods that enhance the mechanical properties of the final parts. A particular interest lies in their strength as it is well known that the strength and stiffness of parts made from 3D-printed thermoplastic materials are not as good as those of parts obtained through injection molding or casting and machining [2,3,4]. The approaches identified in literature can be split into two categories. The first category involves adding extra processing or post-processing steps which have a positive effect on the mechanical properties of final parts. One such effort is the work of Belter et al. [5], who used hollow 3D-printed shells, made from acrylonitrile butadiene styrene (ABS) polymer and infused with thermoset epoxy resin after printing. The technique, named by the authors as “fill compositing”, showed strength increases in the final parts. The authors also showed the use of other resins with different mechanical properties, allowing the printing of parts with functional roles, such as a finger for a robotic hand.

The other category involves mixing or filling a thermoplastic matrix with particles (such as carbon or glass) or short fibers of a different material in order to obtain feedstock filaments with enhanced mechanical characteristics. ABS has been of particular interest for the thermoplastic matrix, as it is a material commonly used in the automotive and consumer goods industries. The introduction of highly oriented chopped carbon fibers in the ABS feedstock results in increased tensile strength and stiffness, according to work done by Tekinalp et al. [6].

While studying these results, one could observe that these techniques have limited applicability and present several disadvantages, such as abrasion of printer nozzle or increased system complexity.

This study looks to assess whether high-strength fibers can be successfully embedded into a thermoplastic substrate during an ME3DP process, by using a pause/resume printing method.

## 2. Materials and Methods

Ultra-high-molecular-weight polyethylene (UHMWPE), also known as high-modulus polyethylene (HMPE), is a subset of thermoplastic polyethylene. Due to its long molecular chains and high molecular mass, loads can be transferred more effectively to the polymer backbone, resulting in a material that is very tough. Out of all the thermoplastics, UHMWPE has the highest impact strength [7]. The long molecular chains also give the material a very low melt flow index, rendering it difficult to be processed through injection/extrusion processes, including ME3DP [8]. For this reason, using the material in the form of pre-made fibers is being investigated. UHMWPE has an ultimate strength of 3.3–3.9 GPa, on par with that of Kevlar and slightly lower than that of carbon fibers. On the other hand, UHMWPE has a low specific gravity of 0.97, making it stronger by weight than all the other fibers, with a strength-to-weight ratio 15 times higher than steel and 40% higher than common aramids. However, one of the big weaknesses of this polymer is its reduced maximum operating temperature. Unlike other strong fibers, such as aramids, that have very high melting points (350 °C for Nomex [9], above 500 °C for Kevlar [10]), UHMWPE has a low melting point of just 130 °C [11,12]. This makes embedding such fibers within a thermoplastic matrix during a 3D printing process difficult, given that commonly used thermoplastic filaments are typically being extruded at temperatures of around 200 °C or more. An alternative is to use a lower melting point polymer, such as polycaprolactone (PCL) [13], with a melting point of just 65 °C. As parts made from PCL have limited uses due to softening at reduced temperatures, polylactic acid (PLA) was considered for this application. PLA has seen a widespread usage in ME3DP due to its processability in a wide temperature range, from 170 °C to 230 °C. PLA has a glass transition temperature of 60 °C and a melting temperature of 160 °C [14]. PLA is cheap to manufacture and procure [15], is biodegradable, and has little-to-no toxicity in its solid form, making it biocompatible [16]. Thus, its applications include biocompatible screws for medical implantation [17], wound dressings using PLA-antioxidant blends [18], and hand splints for emergency splinting [19].

While the material properties of the two components might seem inferior to those of other polymer–fiber composites such as nylon/Kevlar, the properties of the PLA/UHMWPE composite could be seen as an advantage in some areas. In a time when governments and organizations seek to reduce waste and emissions, composites are generally seen as difficult to recycle due to their inherent heterogeneity. According to a review by Yang et al. on recycling of composites [20], there is an increasing demand in easier to recycle composites, which use thermal or mechanical processes to separate and recycle components, as opposed to chemical processes which are hazardous and create unwanted by-products. Important advances have been made in making composites with embedded natural fibers, such as flax fibers [21], bamboo [22,23], or wood flour [24]. The use of natural fibers also has temperature limitations regarding the choice of polymer matrix, as high temperatures induce the thermo-oxidative degradation of bio-fillers [25]. Due to the compostable properties of both PLA and PCL and the chemical resistance of UHMWPE, it is easy to envision a composting process where the matrix materials are composted and long UHMWPE fibers are recovered. Alternatively, a pyrolysis process where materials are decomposed into useful gasses could be used, such as the trash-to-gas process developed in the United States by the National Aeronautics and Space Administration (NASA) [26] to save space and weight during long space missions.

Other potential advantages stem from the physical properties of UHMWPE. Compared to carbon fibers, UHMWPE has higher fracture toughness, allowing it to be formed around tighter radii in a 3D printing process which deposits it as a filament. Kevlar can absorb up to 3.5% of its own weight in water, weakening its mechanical properties, while UHMWPE is non-hygroscopic and has lower wettability even in an etched state.

**Figure 2:**
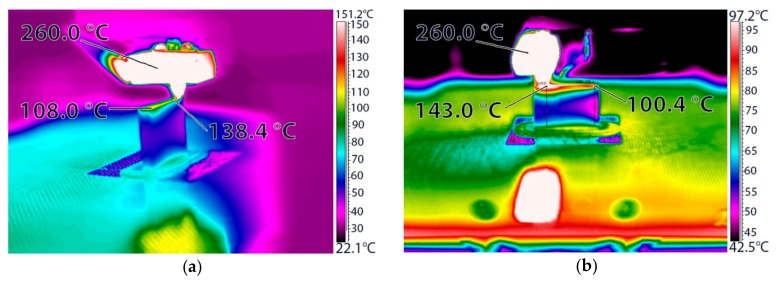
3D printing of an acrylonitrile butadiene styrene and polycarbonate (ABS+PC) thermoplastic material part viewed through a ThermaCAM SC640 (FLIR Systems, Wilsonville, OR, USA) thermographic camera on a Zortrax M200 (Zortrax, Olsztyn, Poland) machine: (**a**) Isometric view; (**b**) side view.

In the ME3DP process, the extruder is heated and then it liquefies the thermoplastic build material, which starts to cool down as soon as it exits the printer nozzle. The cooling of the freshly deposited material happens through convection with the surrounding air and through conduction with the previously deposited material. This behavior is showcased in Figure 2 for an acrylonitrile-butadiene-styrene and polycarbonate (ABS+PC) polymer blend with an extrusion temperature of 260 °C, where it can be observed that even when extruding material with such a high melting point, the temperature of the deposited layer of material quickly drops to temperatures compatible with UHMWPE.

In order to verify whether UHMWPE fibers can be successfully integrated in a 3D-printed product, experimentation started with determining the highest extrusion temperature the fibers can withstand without melting or breaking at the pass of the nozzle. The amount of heat transferred from the thermoplastic material to the fibers varies based on fiber orientation and extrusion pathing. This amount is at its lowest when the nozzle trajectory is perpendicular to the fiber strands and at its highest when they are collinear. Extrusion temperature tests were performed by 3D printing ASTM D638 type I specimens with the printer bed at 40 °C and ambient temperature at 25 °C. Braided Dyneema fibers (DSM, Heerlen, The Netherlands) with diameters of 0.08 mm, 0.10 mm, and 0.16 mm were placed on top of the last deposited PLA layer and roads of filaments were deposited on top of the fibers. The extrusion temperature was set at 170 °C and it was increased in 2 °C increments if fibers did not break or visibly deteriorate. The results of these tests are shown in Section 3.1. of this paper.

The used UHMWPE fibers have a tensile strength of 2.9 GPa (3.3 GPa prior to etching) and a tensile modulus of 120 GPa. Individual filaments composed of these fibers are 12 µm in diameter. The fibers are acquired in a “natural state” (uncoated, not dyed—white color) and subjected to chromic acid surface etching prior to use. The etching treatment consists of submerging the fibers into an etching solution of potassium dichromate (K_2_Cr_2_O_7_), sulfuric acid (H_2_SO_4_), and distilled water in a 7:150:12 mass ratio. After etching for 3 min, the fibers are washed with distilled water, then ethanol, and then they are dried under vacuum. This method is known to increase surface energy and wettability of UHMWPE by increasing roughness and oxidation of the surface, helping improve adhesion characteristics of UHMWPE in polymer matrices [27,28,29].

Literature points to 205–210 °C as the optimal temperature for attaining the maximum strength of PLA 3D-printed parts [30,31]. According to a review by Popescu et al., which investigated the mechanical characteristics of 3D-printed parts depending on various process parameters, the tensile strength of printed parts is influenced by the 3D printer, material supplier, and methodology used to fabricate them [32] and tests should be performed for each specific set-up in order to get accurate results. Thus, in order to assess the feasibility of embedding Dyneema fibers within a PLA matrix and whether this method could improve mechanical properties of the finished 3D-printed parts, tensile strength tests were first performed on unfilled specimens of PLA (FormFutura, Neijmegen, The Netherlands) printed using different extrusion temperature parameters. The fabricated specimens were sized according to ASTM D638 type 1 standard [33] on a QidiTech 3D printer (Zhejiang QIDI Technology, Ruian, China) using a 0.4 mm diameter nozzle. Table 1 shows the process parameters used for manufacturing the specimens.

The specimens were subjected to tensile strength testing on a Hounsfield H10KT universal testing machine (Hounsfield Test Equipment, Redhill, United Kingdom) with a maximum loading capacity of 10 kN. The testing was done with a pre-tensioning force of 5N, at a loading speed of 10 mm/min in conditions of 24°C and 40% humidity. The test results are shown in Section 3.2.

Subsequently, to ensure that the fibers will not melt or break when subjected to the heat of the nozzle and of the deposited thermoplastic material, the minimum extrusion temperature was set at 170 °C. The maximum extrusion temperature was set at 220 °C, which was situated at the higher end of the temperature range provided by the manufacturer.

After concluding that the Dyneema fibers can indeed be embedded in a PLA matrix, albeit at the lower end of the thermoplastic material’s extrusion temperature range, several specimens were manufactured with embedded fibers using the same 3D printer mentioned previously. The specimen type and dimensions were also identical to those fabricated for unfilled specimen testing. The fiber strands layout was chosen according to results reported by Amza et al. [34] for unidirectional glass fiber inserts in a thermoplastic matrix, with three parallel strands of fiber embedded in every second layer of deposited thermoplastic material in order to prevent material agglomeration and the formation of internal voids which would lower the mechanical performance of the resulted parts. Thus, each specimen had a total of 24 strands. The fiber strands were laid manually, after pausing the printing process at the correct layer height. After placing the three strands of fiber longitudinally, the printing process was resumed. This operation was repeated for every second layer of the part. Braided fiber strands of 0.16 mm in diameter were used and four specimens were fabricated using each 3D-printed raster orientation (0° and 45°/−45°).

An unwanted consequence of composite materials manufacturing is the formation of voids due to the inclusion of fibers in the substrate. These voids have the negative effect of increasing material porosity and weakening its mechanical characteristics, and a quantitative assessment is necessary in order to estimate their impact on the composite properties. Due to the nature of the ME3DP process, different geometries of a 3D-printed part impose using different layouts and densities of continuous fiber reinforcement. In many cases, deposited filaments of a thermoplastic cover surfaces of discrete values, as the width of the printed filament is typically a value multiple of extruder nozzle diameter. This means that void formation may not follow a linear increase with fiber density. Thus, a quantitative porosity assessment was made for various levels of UHMWPE fiber density. PLA substrate samples of 50 × 13 × 3.2 mm were 3D printed using the two raster orientations discussed previously and a number of 0 to 6 fibers of 0.16 mm diameter were embedded longitudinally every second layer of the substrate, for a total fiber strand count of 0 to 48 strands. The fibers were evenly spaced. Porosity content for the samples was determined according to ASTM D2734-16 [35]. The weight and volume of the composites were measured according to ASTM D792-13 [36] and the percentage of porosity was calculated based on Equation (1):

(1) $$v_{p}=1-\rho_{c}\left(\frac{1-w_{f}}{\rho_{m}}+\frac{w_{f}}{\rho_{f}}\right)$$

where $v_{p}$ is the porosity content, $\rho_{c}$ is the composite density, $\rho_{m}$ is the matrix density, $\rho_{f}$ is the fiber density, and $w_{f}$ is the fiber weight ratio.

Porosity density testing results are shown in Section 3.3 of this paper.

## 3. Results

### 3.1. Maximum Extrusion Temperature

During testing, fibers with the smallest diameter of 0.08 mm broke under all investigated extrusion temperatures and orientations. It was observed that UHMWPE fibers of 0.16 mm diameter will melt after a pass of the extrusion nozzle at a 45° angle when extrusion temperature was above 186 °C. The tolerance dropped to an extrusion temperature of 174 °C in the case of collinear passes of the extruder relative to fiber direction. While increasing the diameter of fibers did increase the amount of heat required to bring the material to its melting point, in order for the fibers to be embedded properly in the thermoplastic matrix, the height of each deposited thermoplastic layer should be increased as well. This means that the amount of heat deposited by the extruder with each pass would also increase. The results of the temperature compatibility testing are shown in Table 2.

### 3.2. Unfilled PLA Specimens

**Figure 3:**
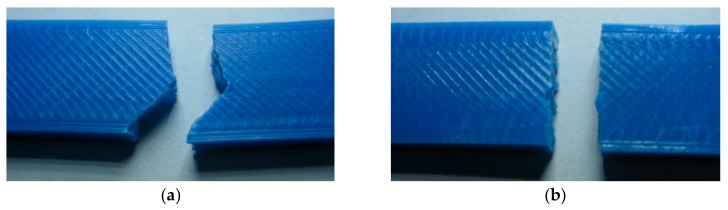
Break pattern of unfilled polylactic acid (PLA) specimens: (**a**) Specimen printed at 170 °C with 45°/−45° raster; (**b**) specimen printed at 220 °C with 45°/−45° raster.

Specimens printed with 45°/−45° raster broke in a zig-zag pattern (Figure 3a), along the filament roads which make up the infill structure, while the specimens printed with 0° raster broke in the transversal plane. In the case of some specimens printed with a 220 °C extrusion temperature and 45°/−45° raster, it was seen (Figure 3b) that the rupture occurred in the same transversal plane, similar to specimens printed with 0° raster, indicating that a much better internal welding of the filament roads had occurred at these increased extrusion temperatures.

**Figure 4:**
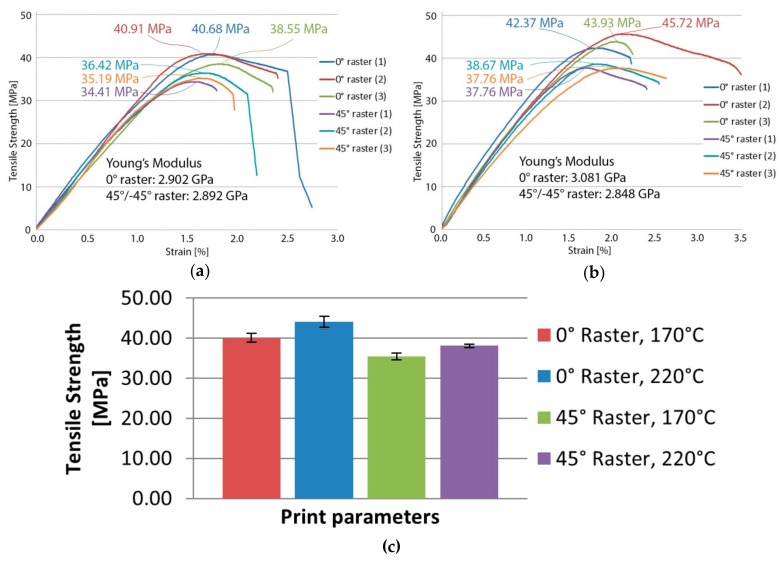
Tensile testing results of unfilled PLA specimens: (**a**) Specimens printed at 170 °C showing an average tensile strength of 40.05 MPa and an average Young’s modulus of 2902 MPa when printed with 0° raster, and a tensile strength of 35.34 MPa and an average Young’s modulus of 2898 MPa for the 45°/−45° raster; (**b**) specimens printed at 220 °C showing an average tensile strength of 44.01 MPa and an average Young’s modulus of 3081 MPa when printed with 0° raster, and a tensile strength of 38.06 MPa and an average Young’s modulus of 2848 MPa for the 45°/−45° raster; (**c**) average tensile strength for unfilled specimens—bars on top of columns represent standard deviation.

Tensile strength tests done on unfilled PLA specimens while varying extrusion temperature showed that the optimal printing temperature for the considered printing parameters and chosen material is 220 °C (Figure 4).

On average, 3D-printed parts with an extrusion temperature at the lower end of PLA’s processability temperature range exhibited worse mechanical properties, tensile strength was reduced by 9% for parts printed with 0° raster and by 7% for parts printed with 45°/−45° raster. This reduction in strength will have to be offset by the UHMWPE fibers, if the PLA/UHMWPE composite is to be feasible. As expected, parts printed with 0° raster performed significantly better than those printed with 45°/−45° raster, due to the deposited material strands forming the horizontal layers aligning with the load.

### 3.3. PLA Specimens Filled with Dyneema Fibers

**Figure 5:**
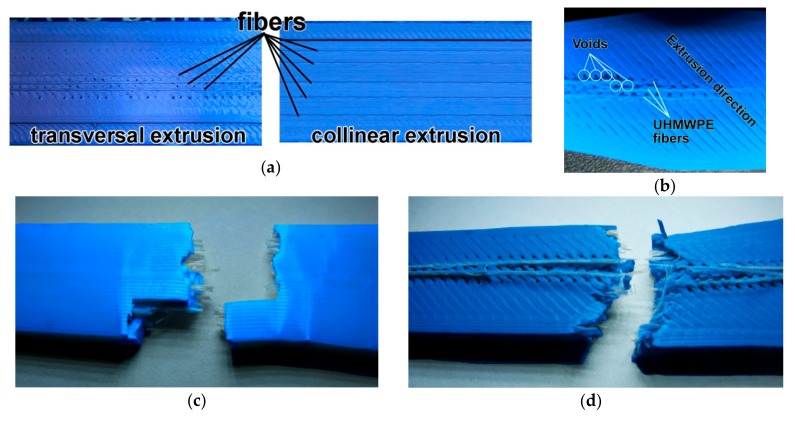
Ultra-high-molecular-weight polyethylene (UHMWPE) fiber layout: (**a**) Fiber direction relative to extrusion pathing; (**b**) void formation in the case of transversal extrusion pathing at a 45° angle; (**c**) specimen rupture for 0° raster; (**d**) specimen rupture for 45°/−45° raster.

The formation of voids due to embedding of fibers negatively affected tensile strength [37] and was most influential in the case of fibers transversal to extrusion pathing (Figure 5b). The effect of the internally created voids is also visible in Figure 5c, as the specimen ruptured along two parallel planes instead of a single plane, such as in the case of unfilled PLA specimens. Fiber breakages with no pull-outs occurred in the case of continuous fibers deposited parallel to thermoplastic filaments and with some fiber pull-outs in the case of specimens with the thermoplastic matrix deposited transversally at a 45° angle. In both cases, the matrix material covered some of the fibers signaling good interaction between the two components.

**Figure 6:**
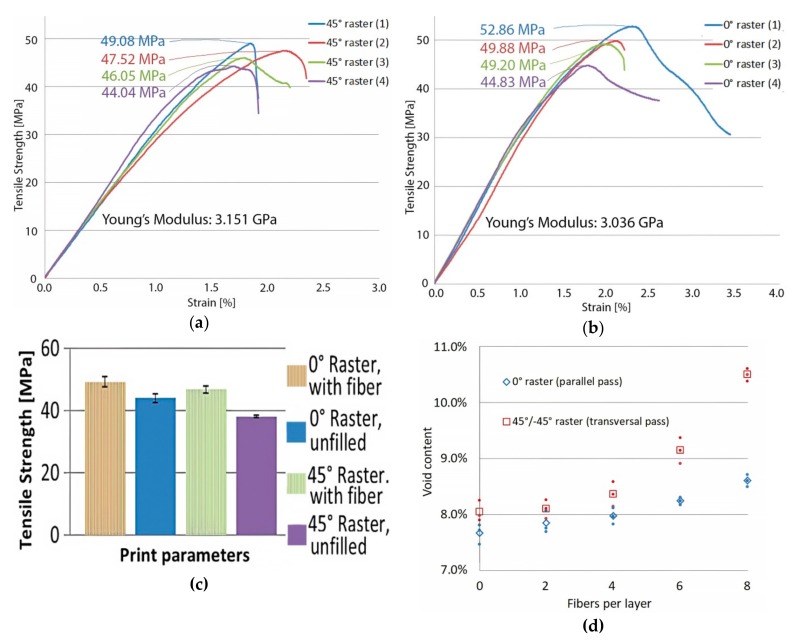
Tensile testing results of PLA specimens filled with unidirectional UHMWPE fibers: (**a**) Specimens printed with a 45°/−45° raster angle showing an average tensile strength of 46.72 MPa and an average Young’s modulus of 3151 MPa; (**b**) specimens printed with a 0° raster angle, showing an average tensile strength of 49.19 MPa and an average Young’s modulus of 3036 MPa; (**c**) average tensile strength for fiber specimens compared to unfilled samples; the bars represent standard deviations; (**d**) void content of test specimens—red square marker represents the average of three 45° samples, rhombic marker represents the average of three 0° raster samples, while round markers represent individual samples. Eight fibers per layer correspond to 2% by weight fiber.

Figure 6a–c shows the tensile strength testing results in the case of the fiber-reinforced specimens. It can be observed that even a small number of fibers (3 strands of 0.16 mm fiber every second substrate layer, 24 strands in total) was sufficient to increase the strength of parts. For parts manufactured with 0° raster, the fiber specimens exhibited a 23% strength increase compared to the unfilled specimen printed with identical process parameters and a 12% net gain relative to the unfilled specimen printed at optimal extrusion temperature. For parts manufactured with 45°/−45° raster, the fiber specimens exhibited a 32% strength increase compared to the unfilled specimen printed with identical process parameters and a 23% net gain relative to the unfilled specimen printed at optimal extrusion temperature.

It is worth mentioning that in the case of fiber-reinforced parts, the average tensile strength was higher than the average tensile strength of unfilled parts, regardless of temperature and raster orientation.

Figure 6d shows the porosity content relative to the number of embedded fibers and deposition path. It can be observed that the embedded fibers increased void formation nearly linearly when PLA deposition was done at 0° raster, while void formation was accentuated at a high fiber count when substrate deposition was done at raster angles of 45°/−45°. The braided architecture of the used UHMWPE fiber had a direct consequence on the porosity content.

**Figure 7:**
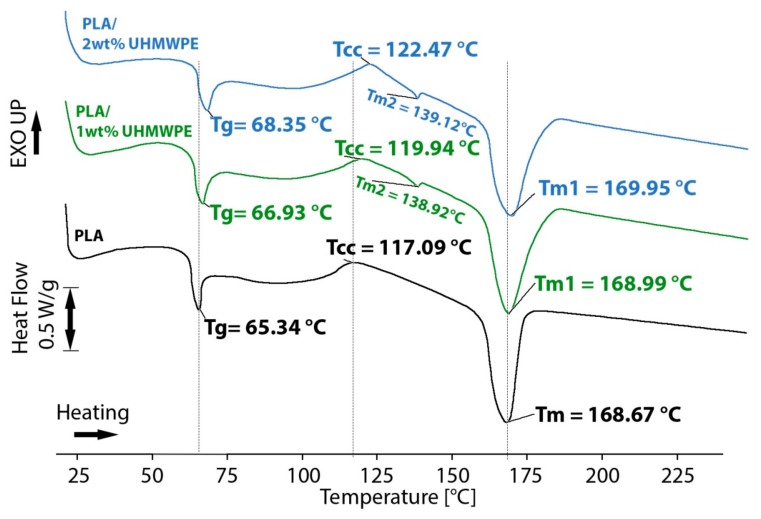
Differential scanning calorimetry (DSC) thermogram (second heating cycle) showing thermal transitions of neat PLA, PLA/1wt% UHMWPE, and PLA/2wt% UHMPWE.

A discussion should also be made regarding the thermal transfer within the newly obtained composite. Unlike composites made with filaments filled with short fibers, which will have an evenly distributed amount of fibers throughout the part and show the same thermal behavior regardless of part geometry, the properties and behavior of a part made with embedded continuous fibers is fundamentally heterogeneous and largely dependent on several geometry and process constraints, such as minimum deposition length, corner radius, and part continuity in the horizontal plane, which restrict the density and length of continuous fiber strands that can be integrated in the structure [38]. For this reason, it is important to highlight that thermal transitions will not show homogenous behavior in a 3D-printed composite with such continuous fibers. A differential scanning calorimetry (DSC) thermogram was performed in order to assess the thermal transitions (glass transition, cold crystallization, and melting) in the composite. Test specimens were manufactured from unfilled PLA, PLA/1wt% UHMWPE fibers (32 strands of 0.16 mm fiber), and PLA/2wt% UHMWPE fibers (64 strands of 0.16 mm fiber). Three specimens of each composition were tested using a Shimadzu DTA-50 machine (Shimadzu Corp., Kyoto, Japan). The first heating cycle was 20–170 °C at a rate of 10 °C/min, followed by cooling down to 30 °C at 5 °C per minute. The second heating cycle was done at 20–240 °C at a rate of 10 °C/min. The analysis was performed in a nitrogen atmosphere. The thermal transitions that occurred during the second heating cycle are shown in Figure 7 and the temperatures at which these transitions occurred are shown in Table 3.

The glass transition temperature of the PLA (*T_g, PLA_* = 65.34 °C) increased slightly for composites with added UHMWPE fibers to *T_g__, 1wt%_* = 66.89 °C for the 1% fiber composite and to *T*g_2wt%_ = 68.35 °C for the 2% fiber composite. The reason for this was that the PLA molecules bonded to the outer surface of the fiber, reducing the mobility of the amorphous fraction in the PLA matrix. Cold crystallization of PLA occurred at *T_cc, PLA_* = 117.09 °C for the neat specimens. When adding reinforcement fibers to the PLA matrix, the interface between the two materials can act as nucleation sites for the crystallization of the matrix polymer or restrict the mobility of the polymer chains. Increasing the fiber content shifted this exothermic transition peak to higher temperatures, with a maximum of *T_cc, 2wt%_* = 122.47 °C and also decreased its peak intensity, indicating that the immobilization effect is more dominant.

The appearance of a second melting peak can be observed at around 139 °C, corresponding to the melting temperature of the UHMWPE fibers. The low mass of fiber content prevented the analysis of UHMWPE crystallinity changes within a sufficiently low error margin.

## 4. Discussion

In order for the fiber compositing process to be successful, the destruction or degradation of the low melting point UHMWPE fibers due to heat from the extruder needs to be prevented. One solution is to reduce the extrusion temperature and use thermoplastic materials that can remain processable at these lowered temperatures. Despite the large range of temperatures, PLA can be processed, and lowering the extrusion temperature to 170 °C causes worsening of mechanical characteristics of the resultant part compared to mechanical properties obtained using optimal printing parameters. Following testing, it was found that embedding strands of high-tensile fibers offers sufficient reinforcement to offset these losses, resulting in a net gain of strength of 12% to 23% depending on raster orientation. These results are remarkable, considering the 24 fiber strands embedded in each specimen represent only 1.16% of the cross-sectional area of the tested specimen. While improved results may be obtained by using a larger number of fiber strands, a tipping point for the strength increase may be reached due to internal defects caused by the addition of fibers. An optimization of the substrate/fiber volume ratios and fiber-laying patterns will need to be performed in the future.

The DSC thermogram analysis shows the distinct melting point of UHMWPE fibers at a temperature of around 139 °C. This is encouraging for the potential of recycling the fibers from the composite, considering the PLA decomposing mechanism. PLA degrades through hydrolysis at temperatures starting with 60 °C into water, CO_2,_ and biomass. These conditions are not expected to affect the mechanical characteristics of the fiber through the recycling process, meaning full-length fibers could be successfully recovered.

While the manual laying of fibers is labor intensive and error prone, automation of the fiber laying process is possible as it is shown in literature [39,40]. A number of techniques currently in the market could be applied to manufacture parts using such composites. One of these techniques is the composite 3D printing process, patented by Markforged (Somerville, MA, USA), which uses continuous carbon fiber or fiberglass strands embedded into a 3D-printed nylon substrate [41,42]. The adaptation of the above-mentioned process for use with the materials described in this paper would start with creating a void-free composite filament with a PCL matrix and UHMWPE fiber core. This is feasible due to the reduced melting temperature of PCL which is insufficient to start degradation of the fiber core. Previous work by Aninyajady et al. describes the possibility of creating a PCL/UHMWPE composite fabric and characterizes the obtained material for use in medical implants [43,44]. The second step would be to use the composite void-free filament in a 3D printer with dual extrusion heads, such as those described in the above-referenced patents, where one of the heating heads brings the composite filament to a temperature past the melting point of the filament’s PCL matrix (65 °C) and deposits it on a pre-calculated path onto the previously-deposited PLA substrate. The interaction and compatibility of the PCL matrix used in the composite filament and the PLA substrate is well documented in literature [45,46,47]. The mechanical characterization of such a composite resulting from automation of the method described here is subject of future research.

## 5. Conclusions

This research aimed at investigating whether ultra-high-molecular-weight polyethylene fibers can be successfully embedded in a thermoplastic matrix during a material extrusion 3D printing process. This is of interest due to the high tensile strength and high creep resistance of UHMWPE fibers. The difficulty of embedding this type of fibers in 3D-printed thermoplastics lies in the relatively low melting temperature of the fibers compared to the usual extrusion temperatures of thermoplastics commonly used in ME3DP processes. Fibers of 0.08, 0.10, and 0.16 mm in diameter have been subjected to the heating regime specific to PLA 3D printing. It was found that small diameter fibers heat up too quickly and melt even at the lowest processable temperature for PLA (170 °C). Fibers of 0.16 mm withstood the printing process up to 186 °C when thermoplastic filaments are deposited at a 45° angle, and up to 174 °C when filaments are deposited in a parallel direction.

Using a manual fiber-laying process, UHMWPE fibers (0.16 mm in diameter) previously etched with chromic acid have been successfully embedded in a PLA matrix. Depending on raster angle and relative orientation of filament deposition paths, the internal void content increases with fiber addition, up to a maximum of 10.5% for specimens made with 45°/−45° raster and up to 8.6% for specimens made with 0° raster at a 2% by weight fiber content. Tensile strength tests on specimens made with a low number of fiber filament reinforcement (1.16% fiber by volume, 0.75% by weight) show an increase in strength of 23% for parts printed with 45°/−45° raster and 12% for parts printed with 0° raster over unfilled specimens printed at the optimal extrusion temperature. The void contents specific to these specimens are 7.9% (0° raster) and 8.2% (45° raster).

The values used for some process parameters such as the quantity and layout of fiber strands have been chosen based on findings presented in other papers from specialty literature in order to prevent material buildup and limit potential errors due to manual handling. While optimizing the mechanical properties of the fibers composite has not been a goal of this paper, it can constitute the body of future work.

Thermal analyses show that the addition of UHWMPE fibers has a small plasticizing effect on the PLA matrix as glass transition of the substrate slightly increased from 65.34 ± 0.32 °C to 68.35 ± 0.50 °C. The fibers have no substantial effect on melting temperatures of the matrix. Future work will analyze from a thermal perspective, the effect of encasing the UHMPWE fibers in a PCL matrix in order to create composite filaments for an automated fiber laying process.

The use of other thermoplastic materials, that have better mechanical properties compared to those of PLA, while still being eco-friendly, as substrates is of future interest. Based on the results shown in Section 3.1, these materials would most likely require additional forced cooling after extrusion to reach a suitable temperature for UHMWPE integration, favoring plastics with low thermal expansion coefficients to prevent cracking, warping, or delamination.

## Figures and Tables

**Table 1:** Print process parameters.

Parameter	Value
Raster angle	0° or 45°/−45°
Specimen cross-section	3.2 mm × 13 mm
Contour width	1.2 mm
Infill	100%

**Table 2:** Results of extrusion temperature testing.

Fiber Diameter (mm)	Layer Height (mm)	Extrusion Path	Maximum Extrusion Temperature (°C)
0.08	0.10/0.20	Collinear/Transversal 45°	N/A ^1^
0.10	0.10	Collinear	N/A
0.10	0.10	Transversal 45°	170
0.10	0.20	Collinear/Transversal 45°	N/A
0.16	0.20	Collinear	174
0.16	0.20	Transversal 45°	186

^1^ Fibers got damaged even at the minimum tested extrusion temperature of 170 °C.

**Table 3:** Thermal transitions of neat PLA and PLA/UHMPWE blends.

Specimen	*T*_g_ (°C)	*T*_cc_ (°C)	*T*_m_ (°C)	Δ*H*_c_ (J/g)	Δ*H*_m_ (J/g)	*X*_c_ (%)
100 PLA	65.34 ± 0.32	117.09 ± 0.27	168.67 ± 0.44	14.33 ± 0.10	34.08 ± 0.12	36.64
99/1 (w/w) PLA/UHMWPE	66.89 ± 0.29	119.94 ± 0.22	168.99 ± 23 (PLA) 138.92 ± 0.14 (UHMWPE)	11.69 ± 0.21	40.01 ± 0.34	43.02
98/2 (w/w) PLA/UHMWPE	68.35 ± 0.50	122.47 ± 0.43	169.95 ± 40 (PLA) 139.12 ± 0.36 (UHMWPE)	11.96 ± 0.19	37.62 ± 0.50	40.45

*T*_g_ is the glass transition temperature; *T*_cc_ is the cold crystallization temperature; *T*_m_ is the melting temperature; Δ*H*_c_ is the enthalpy of crystallization; Δ*H*_m_ is the enthalpy of melting; *X*c is the degree of crystallinity.
